# Identificação pela ultrassonografia vascular dos diâmetros das veias safenas magnas sem refluxo em mulheres

**DOI:** 10.1590/1677-5449.008016

**Published:** 2017

**Authors:** Carlos Alberto Engelhorn, Ana Luiza Engelhorn, Camila Ritter, Gabriel Faria Isfer de Lima, João Gabriel Peixoto Lopes, Letícia Gaertner Cabrini

**Affiliations:** 1 Pontifícia Universidade Católica do Paraná – PUC-PR, Cirurgia Vascular, Curitiba, PR, Brasil.

**Keywords:** veia safena, ultrassonografia, mulheres

## Abstract

**Contexto:**

A ultrassonografia vascular (UV) é o exame de escolha para estudar o sistema venoso superficial dos membros inferiores e mensurar o diâmetro das veias safenas, podendo ser utilizada como parâmetro para o planejamento cirúrgico.

**Objetivos:**

Identificar pela UV os diâmetros de veias safenas magnas sem refluxo em mulheres e sua relação com a idade, altura, Classificação Clínica, Etiologia, Anatomia e Fisiopatologia (CEAP) e índice de massa corporal (IMC).

**Métodos:**

Estudo transversal em mulheres com sintomas de IVC primária (C0, 1 ou 2), sem cirurgia prévia de varizes e sem refluxo detectado pela UV, nas quais foram mensurados os diâmetros da veia safena magna (VSM) na crossa, coxa e perna, que foram comparados com a idade, altura, classe clínica CEAP e IMC.

**Resultados:**

Foram avaliadas 353 mulheres, das quais 146 foram incluídas no estudo sendo 88 avaliadas unilateralmente e 58 bilateralmente. Os diâmetros encontrados para a VSM sem refluxo foram de aproximadamente 6,5 mm na crossa, 4,0 mm na coxa proximal, 3.0 mm na coxa médio-distal e joelho e 2,5 mm na perna. Em todos os segmentos mensurados houve diferença estatisticamente significativa (p < 0,05) na correlação dos diâmetros com IMC. Não houve diferença estatística na correlação da medida dos diâmetros com classe CEAP, altura e idade das pacientes.

**Conclusões:**

Observou-se que os diâmetros de veias safenas magnas sem refluxo independem da classe clínica CEAP 0 ou 1 e 2; da idade e da altura das pacientes. Entretanto, os diâmetros da VSM se relacionam significativamente com o IMC das pacientes.

## INTRODUÇÃO

A insuficiência venosa crônica (IVC) é definida como um estado de anormalidade do funcionamento venoso causada por incompetência valvular, associada ou não à obstrução do fluxo venoso. Tal condição pode afetar o sistema venoso superficial, profundo e as veias perfurantes, devido a distúrbios congênitos ou adquiridos^1^, manifestando-se principalmente com sinais de dor, edema, alterações de pele e ulcerações^2^.

A IVC constitui importante problema de saúde pública e socioeconômico, visto que essa condição é a 14ª maior causa de afastamento temporário do trabalho no Brasil, atingindo aproximadamente 20% da população adulta em países do Ocidente, com 3,6% da população apresentando úlcera venosa^3^.

A ultrassonografia vascular (UV) é o exame de escolha para identificar e localizar obstruções ou refluxos no sistema venoso, além de possibilitar a mensuração do diâmetro das veias safenas, que pode ser utilizado como parâmetro para o planejamento cirúrgico. Contudo, não existem na literatura muitas evidências sobre o calibre normal da veia safena magna (VSM) em mulheres^4^.

Engelhorn et al.^5^ determinaram a relação entre o diâmetro da VSM e a presença de refluxo na junção safeno-femoral (JSF) (> 9 mm), na coxa (> 7 mm) e na perna (> 5 mm). Na ausência de refluxo, o calibre da VSM foi inferior a 5 mm na JSF e 3 mm na coxa. Na perna, no entanto, não foi possível correlacionar a ausência de refluxo com calibres específicos da VSM.

Da mesma forma, ao avaliar a relação entre diâmetro da VSM e presença de refluxo proximal em 182 membros inferiores, Mendoza et al. observaram os valores de 10,5 mm na JSF e 6,2 mm na coxa proximal como preditores de refluxo, sendo a medida da coxa proximal considerada mais sensível e específica para ser utilizada como parâmetro clínico^6^.

O objetivo deste estudo foi identificar pela UV o diâmetro de veias safenas magnas sem refluxo em mulheres e sua relação com idade, altura, classe clínica da Classificação Clínica, Etiologia, Anatomia e Fisiopatologia (CEAP) (C0 a C2) e índice de massa corporal (IMC).

## MÉTODOS

Foi realizado um estudo transversal em mulheres encaminhadas consecutivamente ao laboratório vascular com queixas de IVC para realização do mapeamento venoso pela UV.

Foram incluídas pacientes maiores de 18 anos, com sintomas de IVC primária nas classes clínicas (CEAP) C0, C1 e C2, sem cirurgia prévia de varizes e sem refluxo na veia safena magna detectado pela UV. Foram excluídos homens e foram excluídas mulheres com IVC primária nas classes clínicas (CEAP) C3 a C6, IVC secundária e congênita, safenectomia prévia, tromboflebite recente ou antiga nas veias safenas, e cirurgia bariátrica prévia.

As pacientes foram avaliadas em um laboratório vascular certificado pela ISO 9001 por ultrassonografistas vasculares experientes com certificado de área de atuação pela Sociedade Brasileira de Angiologia e de Cirurgia Vascular (SBACV).

O estudo foi aprovado pelo Comitê de Ética em Pesquisa da Pontifícia Universidade Católica do Paraná, sob protocolo 1.183.464.

No momento do estudo pela UV com as pacientes em posição ortostática, foi realizada a avaliação clínica e classificação (CEAP) de cada um dos membros inferiores (MMII) e foram registrados idade, peso e altura das pacientes para o cálculo do IMC.

### Avaliação ultrassonográfica

As pacientes foram avaliadas com aparelhos Siemens-Antares® e Siemens-X700®, inicialmente para a exclusão de trombose venosa recente ou antiga, em decúbito dorsal, com cortes ultrassonográficos transversais em modo B e manobras de compressibilidade das veias, utilizando transdutores de baixa frequência (5 Mhz).

O estudo da VSM foi realizado com a paciente em posição ortostática, com transdutor de alta freqüência (7-10 Mhz), para a obtenção das imagens das veias em cortes ultrassonográficos transversais em modo B e mensuração dos diâmetros da VSM na crossa, coxa proximal, coxa média, coxa distal, joelho, perna proximal, perna média e perna distal.

Para a análise estatística foi considerado o diâmetro médio da VSM em cada segmento mensurado. A correlação dos diâmetros da VSM com a idade e o IMC foi realizada considerando as classes C0 e C1 *versus* a classe C2, e nos casos com avaliação bilateral da veia safena foi calculada a média dos diâmetros dos dois lados.

Para a comparação de dois grupos em relação ao diâmetro da veia safena, foi usado o teste *t* de Student para amostras independentes. A condição de normalidade das variáveis foi avaliada pelo teste de Kolmogorov-Smirnov. Para a análise da associação entre os diâmetros e outras variáveis quantitativas, foi estimado o coeficiente de correlação de Pearson. Valores de p < 0,05 indicaram significância estatística. Os dados foram analisados com o programa computacional IBM SPSS Statistics v. 20.

## RESULTADOS

Foram incluídas no estudo 146 mulheres com idade variando entre 21 e 79 anos, (média de 45,3 anos), sendo 88 avaliadas unilateralmente e 58 bilateralmente. O total de veias safenas mensuradas foi de 204, sendo 107 (52,5%) do lado direito e 97 (47,5%) do lado esquerdo.

Em relação ao peso e à altura (Tabela 1), as médias foram de 67,1 kg e 1,62 m, respectivamente, com IMC entre 17,5 e 39,5 (média de 25,6).

**Tabela 1:** Valores da idade, peso, altura e IMC.

**Variável**	**n**	**Média**	**Mediana**	**Mínimo**	**Máximo**	**Desvio padrão**
Idade (anos)	146	45,3	44,5	21,0	79,0	13,6
Peso (kg)	146	67,1	65,0	45,0	107,0	11,5
Altura (cm)	146	1,62	1,61	1,49	1,79	0,06
IMC (kg/m^2^)	146	25,6	24,9	17,5	39,5	4,4

IMC = índice de massa corporal.

Dos 204 MMII avaliados, 5 (2,5%) foram classificados como C0, 164 (80,4%) como C1, e 35 (17,2%) como C2.

A média dos diâmetros da VSM (Tabela 2) foi de 6,59 mm na crossa; 4,22 mm na coxa proximal; 3,36 mm na coxa média; 3,13 mm na coxa distal; 3,03 mm no joelho; 2,56 mm na perna proximal; 2,43 mm na perna média e 2,52 mm na perna distal.

**Tabela 2:** Valores dos diâmetros mensurados nos diferentes segmentos da veia safena magna, expressos em mm.

**Segmento**	**n**	**Média**	**Desvio padrão**	**Mediana**	**Mínimo**	**Máximo**	**1º quartil**	**3º quartil**
Crossa	204	6,59	1,36	6,55	3,50	10,20	5,70	7,50
Coxa proximal	204	4,22	1,02	4,10	2,00	7,50	3,50	4,80
Coxa média	204	3,36	0,73	3,30	1,90	5,60	2,80	3,85
Coxa distal	204	3,13	0,74	3,00	1,60	5,80	2,60	3,60
Joelho	204	3,03	0,71	2,95	1,50	5,30	2,50	3,50
Perna proximal	204	2,56	0,61	2,50	1,20	4,40	2,10	3,00
Perna média	204	2,43	0,56	2,40	1,00	4,10	2,00	2,90
Perna distal	204	2,52	0,57	2,50	1,20	4,10	2,20	2,90

**Figura 1:**
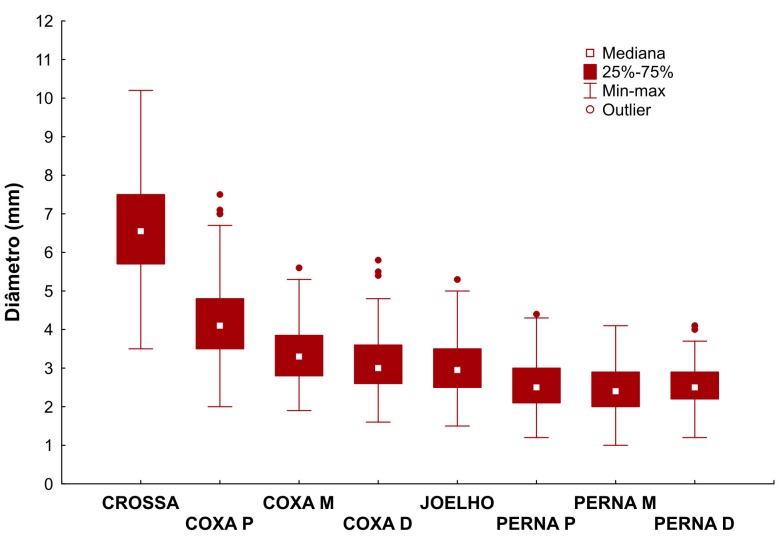
Médias dos diâmetros nos diferentes segmentos da veia safena magna mensurados.

Para cada segmento da VSM mensurado, considerando as classes clínicas C0 ou C1 *versus* C2 (Tabela 3), observou-se que, com exceção da perna proximal, não houve diferença estatisticamente significativa nos diâmetros entre as classes clínicas avaliadas. As médias e medianas dos diâmetros da veia safena magna nos diferentes segmentos mensurados podem ser observadas nas Figuras 1 e 2.

**Tabela 3:** Comparação dos diâmetros em diferentes segmentos da veia safena magna nas classes clínicas C0 e C1 *versus* C2 da classificação CEAP, expressos em mm.

**Segmento**	**Classificação CEAP**	**n**	**Média**	**Mediana**	**Mínimo**	**Máximo**	**Desvio padrão**	**Valor de p** ^*^
Crossa	0 ou 1	169	6,52	6,50	3,50	10,20	1,32	
	2	35	6,94	6,90	4,00	9,50	1,48	0,094
Coxa proximal	0 ou 1	169	4,18	4,10	2,00	7,10	1,02	
	2	35	4,43	4,40	2,50	7,50	1,01	0,190
Coxa média	0 ou 1	169	3,32	3,30	1,90	5,00	0,70	
	2	35	3,54	3,30	2,20	5,60	0,85	0,110
Coxa distal	0 ou 1	169	3,11	3,00	1,60	5,80	0,71	
	2	35	3,23	3,10	1,80	5,50	0,84	0,414
Joelho	0 ou 1	169	2,99	2,90	1,50	5,00	0,69	
	2	35	3,19	3,00	1,60	5,30	0,77	0,136
Perna proximal	0 ou 1	169	2,52	2,50	1,30	4,30	0,60	
	2	35	2,77	2,90	1,20	4,40	0,61	0,024
Perna média	0 ou 1	169	2,40	2,40	1,20	3,80	0,54	
	2	35	2,56	2,60	1,00	4,10	0,61	0,121
Perna distal	0 ou 1	169	2,50	2,40	1,20	4,10	0,57	
	2	35	2,63	2,60	1,20	4,00	0,58	0,193

* Teste *t* de Student para amostras independentes, p < 0,05.

**Figura 2:**
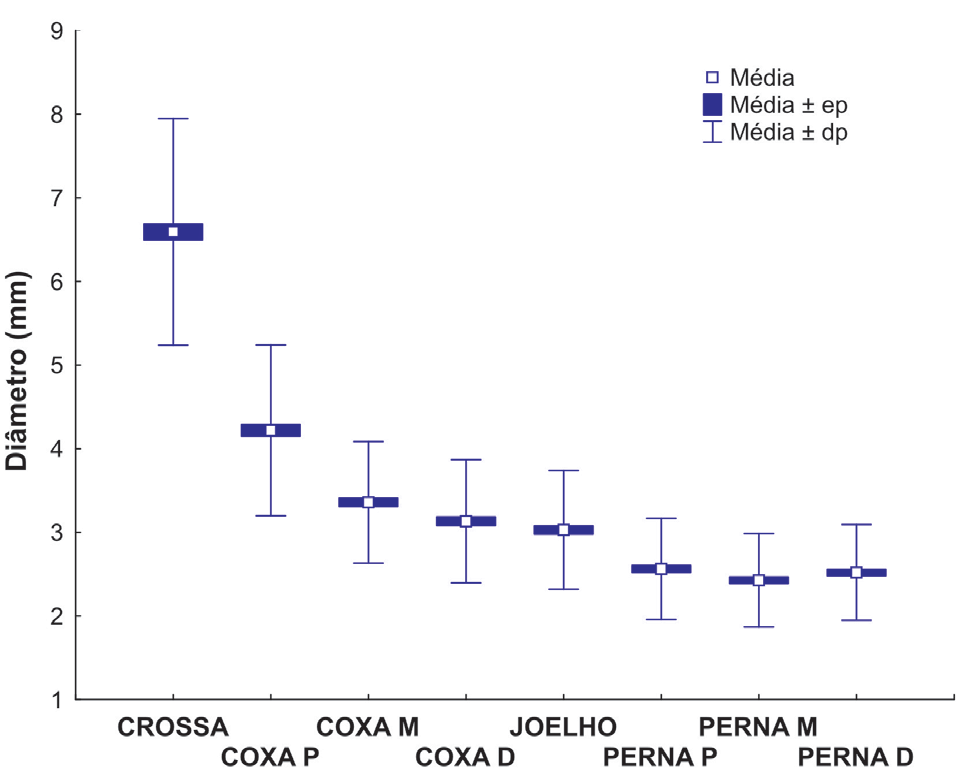
Medianas dos diâmetros nos diferentes segmentos da veia safena magna mensurados.

Em relação à associação entre o IMC das pacientes e o diâmetro da VSM nos diversos segmentos mensurados (Tabela 4), apesar de o coeficiente de correlação variar entre 0,23 e 0,38, foi observada diferença estatisticamente significativa (p < 0,05) em todos os segmentos avaliados, demonstrando que, quanto maior o IMC, maiores são os diâmetros em toda a extensão da VSM sem refluxo.

**Tabela 4:** Associação entre os diâmetros da veia safena magna e IMC.

**Variáveis**	**n**	**Coeficiente de correlação de Pearson**	**Valor de p**
IMC x crossa	146	0,34	< 0,001
IMC x coxa proximal	146	0,38	< 0,001
IMC x coxa média	146	0,26	0,001
IMC x coxa distal	146	0,23	0,005
IMC x joelho	146	0,25	0,002
IMC x perna proximal	146	0,30	< 0,001
IMC x perna média	146	0,28	0,001
IMC x perna distal	146	0,23	0,004

IMC: índice de massa corporal.

**Figura 3:**
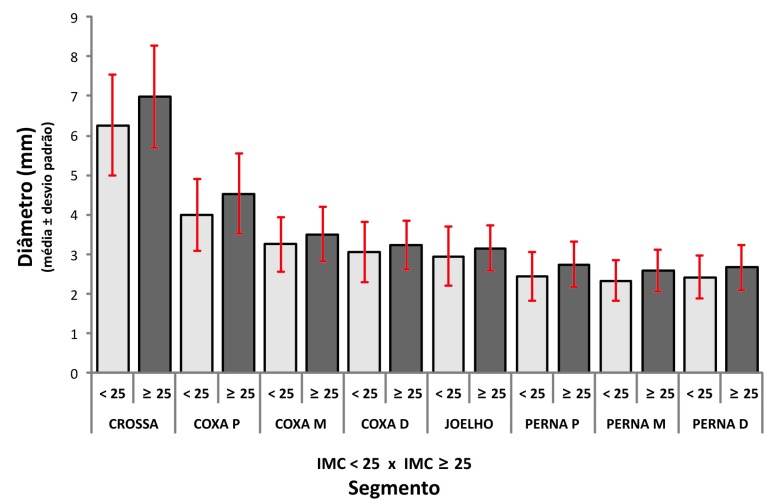
Comparação entre os diâmetros da veia safena magna nos grupos com índice de massa corporal < 25 e ≥ 25.

Ainda com relação ao IMC, considerando o valor médio de 25, foi realizada a comparação entre as pacientes com IMC < 25 e ≥ 25 (Tabela 5), e foi observado que, com exceção do segmento distal de coxa e joelho, houve diferença estatisticamente significativa nas mulheres com IMC ≥ 25 (Figura 3), ou seja, mulheres com IMC ≥ 25 apresentam diâmetros maiores em quase toda a extensão da VSM.

**Tabela 5:** Comparação entre os diâmetros da veia safena magna nos grupos com IMC < 25 e ≥ 25.

**Segmento**	**IMC**	**n**	**Média**	**Mediana**	**Mínimo**	**Máximo**	**Desvio padrão**	**Valor de p** ^*^
Crossa	< 25	75	6,26	6,20	3,50	9,70	1,27	
	≥ 25	71	6,99	7,00	4,20	10,20	1,30	0,001
Coxa proximal	< 25	75	3,99	4,00	2,15	7,50	0,91	
	≥ 25	71	4,53	4,55	2,65	6,70	1,01	0,001
Coxa média	< 25	75	3,25	3,20	1,90	5,00	0,68	
	≥ 25	71	3,50	3,50	2,30	5,00	0,69	0,027
Coxa distal	< 25	75	3,06	3,00	1,75	5,80	0,77	
	≥ 25	71	3,24	3,30	2,00	4,40	0,62	0,128
Joelho	< 25	75	2,94	2,80	1,60	5,30	0,75	
	≥ 25	71	3,15	3,10	2,00	4,60	0,57	0,060
Perna proximal	< 25	75	2,44	2,40	1,20	3,80	0,61	
	≥ 25	71	2,74	2,70	1,40	4,40	0,57	0,002
Perna média	< 25	75	2,33	2,30	1,00	3,50	0,51	
	≥ 25	71	2,59	2,70	1,30	3,80	0,53	0,003
Perna distal	< 25	75	2,42	2,40	1,20	3,60	0,54	
	≥ 25	71	2,66	2,65	1,60	4,10	0,57	0,011

* Teste *t* de Student para amostras independentes, p < 0,05. IMC = índice de massa corporal.

Em relação à idade das pacientes e o diâmetro da VSM (Tabela 6), não houve diferença estatisticamente significativa em todos os segmentos mensurados.

**Tabela 6:** Associação entre os diâmetros da veia safena magna e idade das pacientes

**Variáveis**	**n**	**Coeficiente de correlação de Pearson**	**Valor de p**
Idade x crossa	146	0,16	0,059
Idade x coxa proximal	146	0,13	0,127
Idade x coxa média	146	0,09	0,297
Idade x coxa distal	146	0,05	0,578
Idade x joelho	146	-0,01	0,940
Idade x perna proximal	146	0,13	0,130
Idade x perna média	146	0,17	0,045
Idade x perna distal	146	0,13	0,111

Além do IMC e da idade, a altura e o peso das pacientes também foram comparados com o diâmetro da VSM, sem que fossem observadas diferenças estatisticamente significativas.

## DISCUSSÃO

A UV estuda a anatomia e hemodinâmica do sistema venoso, permitindo o planejamento cirúrgico individualizado de cada extremidade. Além dos padrões de refluxo nas veias safenas^7^, a mensuração dos diâmetros pode ser utilizada como parâmetro na decisão de realizar safenectomias ou procedimentos endovasculares.

Com o objetivo de relacionar o refluxo venoso e o diâmetro de diferentes segmentos da veia safena magna, Engelhorn et al.^5^ estudaram uma amostra de 100 MMII predominantemente em mulheres e determinaram que veias com calibre superior a 7 mm na JSF apresentaram maior chance de refluxo, com acurácia de 71% e valor preditivo positivo de 73%. Para calibres maiores que 4 mm na coxa, a acurácia foi de 75% e o valor preditivo positivo foi de 81%; para calibres maiores que 4 mm na perna, a acurácia foi de 74% e o valor preditivo positivo foi de 89%.

O objetivo deste trabalho foi identificar pela UV o diâmetro de diferentes segmentos de veias safenas magnas sem refluxo exclusivamente em mulheres, devido à maior prevalência da IVC nesse sexo e à possibilidade de o diâmetro ser utilizado como parâmetro para a tomada de decisão terapêutica. Além disso, existe a necessidade de preservação da VSM como uma opção para pontes cardíacas ou periféricas e para técnicas de tratamento endovascular para as varizes dos MMII^7^.

Existem poucas evidências na literatura sobre o calibre da VSM sem refluxo em mulheres. A média dos diâmetros da veia safena neste estudo foi de: 6,59 mm na crossa; 4,22 mm na coxa proximal; 3,36 mm na coxa média; 3,13 mm na coxa distal; 3,03 mm no joelho; 2,56 mm na perna proximal; 2,43 mm na perna média e 2,52 mm na perna distal.

Além de pesquisar os diâmetros normais nas veias safenas magnas, correlacionamos esses diâmetros com idade, altura e IMC das pacientes. Seidel et al.^8^, também utilizando a UV, avaliaram 52 membros inferiores de 26 voluntários (seis homens e 20 mulheres) sem sinais clínicos de IVC e compararam os diâmetros médios da VSM com o IMC de cada indivíduo, sem encontrar diferença estatisticamente significativa.

Em nosso estudo, ao correlacionarmos os valores médios dos diâmetros nos diversos segmentos da VSM com o IMC das pacientes examinadas, observamos que, apesar de uma fraca correlação, houve diferença estatisticamente significativa entre esses achados, demonstrando uma tendência de que, quanto maior o IMC, maior o diâmetro médio em quase toda a extensão da VSM.

Considerando o valor médio do IMC de 25 nas pacientes estudadas e comparando tal valor com as médias dos diâmetros entre as pacientes com IMC ≥ 25 e IMC < 25, foi observado que, excetuando os segmentos distal de coxa e joelho, houve diferença significativa entre esses grupos de pacientes.

Se considerarmos isoladamente a altura e o peso das pacientes, não houve diferença estatisticamente significativa entre todos os segmentos mensurados da VSM.

Kröger et al.^9^ calcularam a área de segmentos da VSM em um estudo transversal com homens e mulheres em duas cidades alemãs. Como a área de um círculo é diretamente proporcional ao diâmetro, a comparação com nosso estudo é pertinente. Os autores demonstraram que não houve relação significativa entre a idade dos pacientes e o aumento do diâmetro na VSM, principalmente em pacientes classificados em C0. Além disso, concluíram também que o aumento do IMC é o fator mais importante para o aumento da área, corroborando com os resultados encontrados nesta pesquisa.

Em nosso estudo, avaliamos a relação entre os diâmetros da VSM e a idade das pacientes, uma vez que a prevalência da IVC tende a aumentar com a idade. Capitão et al.^10^ mostram em um estudo epidemiológico de IVC realizado em Portugal que a IVC grau 3 aumenta significativamente a partir dos 50 anos de idade, independentemente do sexo. Pela necessidade do nosso estudo de avaliar veias safenas sem refluxo, foram incluídos pacientes sem doença venosa avançada (classes clínicas C0 a C2), e nessa população específica observamos que VSM normais não se alteram com a idade nos indivíduos com IVC leve a moderada.

Em conclusão, os autores identificaram diâmetros para a VSM sem refluxo de aproximadamente 6,5 mm na crossa, 4,0 mm coxa proximal, 3.0 mm na coxa médio-distal e joelho, e 2,5 mm na perna. Além disso, concluíram que esses diâmetros independem da classe clínica CEAP 0 ou 1 e 2, da idade e da altura, mas estão relacionados com o IMC das pacientes.
