# Supplementary material for: Identificação pela ultrassonografia vascular dos diâmetros das veias safenas magnas sem refluxo em mulheres
Source: J Vasc Bras. 2017 Apr-Jun;16(2):92–7. [Article in Portuguese] doi: 10.1590/1677-5449.008016 (PMC5915856; doi:10.1590/1677-5449.008016)
Supplement: Translation

# Translation: Vascular ultrasonographic measurement of diameters of great saphenous veins without reflux in women

**Authors:** Carlos Alberto Engelhorn, Ana Luiza Engelhorn, Camila Ritter, Gabriel Faria Isfer de Lima, João Gabriel Peixoto Lopes, Letícia Gaertner Cabrini

**Affiliations:** 1 Pontifícia Universidade Católica do Paraná – PUC-PR, Cirurgia Vascular, Curitiba, PR, Brazil.

**Keywords:** saphenous vein, ultrasonography, women

## Abstract

**Background:**

Vascular ultrasonography (VU) is the examination of choice for studying the superficial venous system of the lower limbs and using VU to measure the diameters of saphenous veins could provide parameters for planning surgery.

**Objectives:**

To employ VU to identify the diameters of great saphenous veins free from reflux in women and determine their relationships with age, height, CEAP classification, and body mass index (BMI).

**Methods:**

This was a cross-sectional study in women with symptoms of primary chronic venous insufficiency (CEAP C0, 1, or 2) with no previous varicose vein surgery and no reflux detected by VU. The diameters of great saphenous veins (GSV) at the junction, thigh, and leg were measured with VU and correlated with age, height, CEAP clinical classification, and BMI.

**Results:**

We assessed 204 limbs in 146 women. The GSV diameters measured were 6.5 mm at the saphenofemoral junction, 4.0 mm at the proximal thigh, 3.0 mm at the mid thigh, distal thigh, and knee and 2.5 mm at the leg. In all segments measured, there were statistically significant differences (p<0.05) when diameters were correlated with BMI. There were no statistically significant differences when diameters were correlated with CEAP class, height, or age.

**Conclusions:**

We observed that the diameters of great saphenous veins free from reflux were independent of CEAP clinical classes 0/1 or 2; age; and height. However, GSV diameters were significantly related to patients’ BMI.

## INTRODUCTION

Chronic venous insufficiency (CVI) is defined as an abnormal state of venous function caused by valve incompetence, which may or may not be associated with obstruction of venous flow. The condition can affect the superficial and deep vein systems and perforating veins, may be due to congenital or acquired disorders,^1^ and primarily manifests with signs of pain, edema, skin disorders, and ulceration.^2^

Chronic venous insufficiency is an important problem both for public health and socioeconomically, since it is the 14th-ranked cause of temporary absence from work in Brazil, affecting approximately 20% of the adult population in Western countries, where 3.6% of the population have venous ulcers.^3^

Vascular ultrasonography (VU) is the examination technique of choice for identifying and locating obstructions or reflux in the venous system, and also offers the possibility of measuring the diameters of saphenous veins, which can be used as parameters when planning surgery. However, there is little evidence in the literature on the normal caliber of the great saphenous vein (GSV) in women.^4^

Engelhorn et al. determined a relationship between presence of reflux and diameters of the GSV at the saphenofemoral junction (SFJ) (> 9 mm), the thigh (> 7 mm), and the leg (> 5 mm). In the absence of reflux, the caliber of the GSV was below 5 mm at the SFJ and < 3 mm at the thigh. However, in the leg it was not possible to correlate absence of reflux with specific GSV calibers.^5^

Along the same lines, Mendoza et al.^6^ investigated the relationship between GSV diameter and presence of proximal reflux in 182 lower limbs, observing that values of 10.5 mm at the SFJ and 6.2 mm at the proximal thigh were predictors of reflux, while the proximal thigh measurement was considered more sensitive and specific for use as a clinical parameter.

The objective of this study was to use VU to measure the diameters of great saphenous veins free from reflux, in women, and their relationships with age, height, clinical class according to the Clinical, Etiological, Anatomical, and Pathophysiological (CEAP) system (C0 to C2), and body mass index (BMI).

## METHODS

A cross-sectional study was conducted of women referred consecutively to a vascular laboratory with CVI-related complaints for venous mapping with VU.

The patients recruited were over the age of 18, had symptoms of primary CVI in (CEAP) clinical classes C0, C1, or C2, had not had prior surgery for varicose veins, and were free from reflux in the great saphenous vein, according to VU examination. Men were not recruited and women were excluded if they had primary CVI in (CEAP) clinical classes C3 to C6, secondary and congenital CVI, prior saphenectomies, recent or historic thrombophlebitis of saphenous veins, or prior bariatric surgery.

Patients were assessed in a vascular laboratory certified to ISO 9001 by experienced vascular ultrasonographers, certified in their areas of expertise by the Brazilian Angiology and Vascular Surgery Society (Sociedade Brasileira de Angiologia e de Cirurgia Vascular).

The study was approved by the Research Ethics Committee at the Pontifícia Universidade Católica do Paraná, under protocol number 1.183.464.

During the VU examination, with patients in a standing position, a clinical assessment and classification (CEAP) was conducted of each lower limb and patients’ age, weight, and height were recorded, for calculation of BMI.

### Ultrasonographic assessment

Patients were examined using Siemens-Antares® or Siemens-X700®, initially to rule out recent or chronic venous thrombosis, in decubitus dorsal, with transverse ultrasonographic sweeps in mode B and vein compressibility maneuvers, using a low frequency transducer (5 Mhz).

The GSV was studied with the patient standing upright, with a high-frequency transducer (7-10 Mhz) to obtain transverse ultrasound images of the vein in mode B, measuring the diameter of the GSV at the SFJ, proximal thigh, mid thigh, distal thigh, knee, proximal leg, mid leg, and distal leg.

The mean GSV diameter at each segment was used for the purposes of statistical analysis. Correlations between GSV diameters and age and BMI were analyzed by comparing classes C0 and C1 against class C2, and in cases in which the saphenous veins were examined bilaterally, the mean diameter of both sides was calculated.

Student’s *t* test for independent samples was used to compare two groups in terms of saphenous vein diameter. Normality of variables was tested using the Kolmogorov-Smirnov test. Pearson’s correlation coefficients were estimated to analyze associations between diameters and other quantitative variables. Results with p < 0.05 were defined as statistically significant. Data were analyzed with IBM SPSS Statistics v. 20.

## RESULTS

A sample was recruited comprising 146 women with ages ranging from 21 to 79 years (mean: 45.3 years), 88 of whom were assessed unilaterally and 58 bilaterally. The total number of saphenous veins measured was 204: 107 (52.5%) on the right side and 97 (47.5%) on the left side.

Mean weight and height (Table 1) were 67.1 kg and 1.62 m respectively, and BMI ranged from 17.5 to 39.5 (mean of 25.6).

**Table 1:** Patients’ age, weight, height, and BMI.

**Variable**	**n**	**Mean**	**Median**	**Minimum**	**Maximum**	**Standard deviation**
Age (years)	146	45.3	44.5	21.0	79.0	13.6
Weight (kg)	146	67.1	65.0	45.0	107.0	11.5
Height (cm)	146	1.62	1.61	1.49	1.79	0.06
BMI (kg/m^2^)	146	25.6	24.9	17.5	39.5	4.4

BMI = body mass index.

Five of the total of 204 lower limbs assessed (2.5%) were classified as C0, 164 (80.4%) as C1, and 35 (17.2%) as C2.

Mean GSV diameters (Table 2) were 6.59 mm at the SFJ; 4.22 mm at the proximal thigh; 3.36 mm at the mid thigh; 3.13 mm at the distal thigh; 3.03 mm at the knee; 2.56 mm at the proximal leg; 2.43 mm at the mid leg; and 2.52 mm at the distal leg.

**Table 2:** Values of diameters measured at different segments of the great saphenous vein, expressed in mm.

**Segment**	**n**	**Mean**	**Standard deviation**	**Median**	**Minimum**	**Maximum**	**1st quartile**	**3rd quartile**
SFJ	204	6.59	1.36	6.55	3.50	10.20	5.70	7.50
Proximal thigh	204	4.22	1.02	4.10	2.00	7.50	3.50	4.80
Mid thigh	204	3.36	0.73	3.30	1.90	5.60	2.80	3.85
Distal thigh	204	3.13	0.74	3.00	1.60	5.80	2.60	3.60
Knee	204	3.03	0.71	2.95	1.50	5.30	2.50	3.50
Proximal leg	204	2.56	0.61	2.50	1.20	4.40	2.10	3.00
Mid leg	204	2.43	0.56	2.40	1.00	4.10	2.00	2.90
Distal leg	204	2.52	0.57	2.50	1.20	4.10	2.20	2.90

SFJ = saphenofemoral junction.

With the exception of the proximal leg measurements, there were no statistically significant differences between clinical classes C0/C1 and C2 for any of the GSV segments assessed (Table 3). Figures 1 and 2 illustrate mean and median diameters of the great saphenous veins at the different segments measured.

**Table 3:** Comparison of diameters of several segments of the great saphenous vein in CEAP clinical classes C0/C1 vs. C2, expressed in mm.

**Segment**	**CEAP classification**	**n**	**Mean**	**Median**	**Minimum**	**Maximum**	**Standard deviation**	**p** ^*^
SFJ	0 or 1	169	6.52	6.50	3.50	10.20	1.32	
	2	35	6.94	6.90	4.00	9.50	1.48	0.094
Proximal thigh	0 or 1	169	4.18	4.10	2.00	7.10	1.02	
	2	35	4.43	4.40	2.50	7.50	1.01	0.190
Mid thigh	0 or 1	169	3.32	3.30	1.90	5.00	0.70	
	2	35	3.54	3.30	2.20	5.60	0.85	0.110
Distal thigh	0 or 1	169	3.11	3.00	1.60	5.80	0.71	
	2	35	3.23	3.10	1.80	5.50	0.84	0.414
Knee	0 or 1	169	2.99	2.90	1.50	5.00	0.69	
	2	35	3.19	3.00	1.60	5.30	0.77	0.136
Proximal leg	0 or 1	169	2.52	2.50	1.30	4.30	0.60	
	2	35	2.77	2.90	1.20	4.40	0.61	0.024
Mid leg	0 or 1	169	2.40	2.40	1.20	3.80	0.54	
	2	35	2.56	2.60	1.00	4.10	0.61	0.121
Distal leg	0 or 1	169	2.50	2.40	1.20	4.10	0.57	
	2	35	2.63	2.60	1.20	4.00	0.58	0.193

* Student’s *t* test for independent samples, p < 0.05. SFJ = saphenofemoral junction.

**Figure 1:** Mean diameters of measured at different segments of the great saphenous vein.

**Figure 2:** Median diameters measured at different segments of the great saphenous vein.

In contrast, although the correlation coefficients for associations between patients’ BMI and the diameter of the GSV at the different segments measured (Table 4) varied from 0.23 to 0.38, tests revealed statistically significant differences (p < 0.05) for all segments, showing that the higher the BMI, the larger the diameter of the entire extension of GSVs without reflux.

**Table 4:** Associations between diameters of great saphenous vein and BMI.

**Variables**	**n**	**Pearson’s correlation coefficient**	**p**
BMI x SFJ	146	0.34	< 0.001
BMI x proximal thigh	146	0.38	< 0.001
BMI x mid thigh	146	0.26	0.001
BMI x distal thigh	146	0.23	0.005
BMI x knee	146	0.25	0.002
BMI x proximal leg	146	0.30	< 0.001
BMI x mid leg	146	0.28	0.001
BMI x distal leg	146	0.23	0.004

BMI = body mass index, SFJ = saphenofemoral junction.

Still with relation to BMI, considering the mean value of 25, a comparison between patients with BMI < 25 and those with BMI ≥ 25 (Table 5) showed that, with the exception of the distal thigh and the knee, there was a statistically significant difference between women with BMI < 25 and ≥ 25 (Figure 3). Women with BMI ≥ 25 had larger diameters along almost the entire extension of the GSV.

**Table 5:** Comparison between diameters of great saphenous vein in groups with body mass index < 25 and ≥ 25.

**Segment**	**BMI**	**n**	**Mean**	**Median**	**Minimum**	**Maximum**	**Standard deviation**	**p** ^*^
SFJ	< 25	75	6.26	6.20	3.50	9.70	1.27	
	≥ 25	71	6.99	7.00	4.20	10.20	1.30	0.001
Proximal thigh	< 25	75	3.99	4.00	2.15	7.50	0.91	
	≥ 25	71	4.53	4.55	2.65	6.70	1.01	0.001
Mid thigh	< 25	75	3.25	3.20	1.90	5.00	0.68	
	≥ 25	71	3.50	3.50	2.30	5.00	0.69	0.027
Distal thigh	< 25	75	3.06	3.00	1.75	5.80	0.77	
	≥ 25	71	3.24	3.30	2.00	4.40	0.62	0.128
Knee	< 25	75	2.94	2.80	1.60	5.30	0.75	
	≥ 25	71	3.15	3.10	2.00	4.60	0.57	0.060
Proximal leg	< 25	75	2.44	2.40	1.20	3.80	0.61	
	≥ 25	71	2.74	2.70	1.40	4.40	0.57	0.002
Mid leg	< 25	75	2.33	2.30	1.00	3.50	0.51	
	≥ 25	71	2.59	2.70	1.30	3.80	0.53	0.003
Distal leg	< 25	75	2.42	2.40	1.20	3.60	0.54	
	≥ 25	71	2.66	2.65	1.60	4.10	0.57	0.011

* Student’s *t* test for independent samples, p < 0.05. BMI = body mass index, SFJ = saphenofemoral junction.

**Figure 3:** Comparison between diameters of great saphenous vein in groups with body mass index < 25 and ≥ 25.

There was no statistically significant relationship between age of patients and GSV diameter at any of the segments measured (Table 6).

**Table 6:** Associations between diameters of great saphenous vein and patient age.

**Variables**	**n**	**Pearson’s correlation coefficients**	**p**
Age x SFJ	146	0.16	0.059
Age x proximal thigh	146	0.13	0.127
Age x mid thigh	146	0.09	0.297
Age x distal thigh	146	0.05	0.578
Age x knee	146	-0.01	0.940
Age x proximal leg	146	0.13	0.130
Age x mid leg	146	0.17	0.045
Age x distal leg	146	0.13	0.111

SFJ = saphenofemoral junction

In addition to BMI and age, patients’ height and weight were also compared in relation to GSV diameter, but no statistically significant differences were observed.

## DISCUSSION

Vascular ultrasonography can be used to study the anatomy and hemodynamics of the venous system, allowing surgical planning to be tailored to each extremity. In addition to patterns of reflux in the saphenous veins,^7^ measurement of vein diameters can be used as a parameter in decisions on whether to conduct saphenectomies or endovascular procedures.

Engelhorn et al.^5^ studied a sample of 100 lower limbs, predominantly in women, with the objective of relating venous reflux to the diameters of different segments of the great saphenous vein. They determined that veins with caliber greater than 7 mm at the SFJ exhibited greater chances of reflux, with an accuracy of 71% and a positive predictive value of 73%. For calibers larger than 4 mm at the thigh, accuracy was 75% and the positive predictive value was 81%; for calibers greater than 4 mm at the leg, accuracy was 74% and the positive predictive value was 89%.

The objective of this study was to use VU to identify the diameters of different segments of great saphenous veins free from reflux, exclusively in women because of the higher prevalence of CVI in females, and in view of the possibility that diameter could be used as a parameter in treatment decision-making. Additionally, there is a need to preserve the GSV as an option for cardiac or peripheral bypass and for endovascular varicose vein treatment techniques.^7^

There is little evidence available in the literature on the caliber of GSVs without reflux in women. The mean diameters of the saphenous vein observed in this study were: 6.59 mm at the SFJ; 4.22 mm at the proximal thigh; 3.36 mm at the mid thigh; 3.13 mm at the distal thigh; 3.03 mm at the knee; 2.56 mm at the proximal leg; 2.43 mm at the mid leg; and 2.52 mm at the distal leg.

In addition to investigating normal diameters of the great saphenous vein, we also correlated these diameters with patients’ age, height, and BMI. Seidel et al. also used VU to assess 52 lower limbs in 26 volunteers (six men and 20 women) without clinical signs of CVI and compared mean GSV diameters with each patient’s BMI, without detecting any statistically significant differences.^8^

In our study, when we correlated mean values of diameters of the different segments of the GSV with the BMI of the patients examined, we observed that, although the correlation was weak, there was a statistically significant difference between findings, demonstrating a tendency that the higher the BMI, the greater the mean diameter along almost the entire extension of the GSV.

Considering the mean BMI value of 25 in the patients studied and using that value to compare mean diameters between patients with BMI ≥ 25 and patients with BMI < 25, it was observed that, with the exception of the distal thigh and the knee, there was a statistically significant difference between these groups of patients.

Taken in isolation, patients’ height and weight were not related to any statistically significant difference in any of the GSV segments measured.

Kröger et al.^9^ calculated the area of GSV segments in a cross-sectional study with men and women conducted in two cities in Germany. Since the area of a circle is directly proportional to its diameter, comparison with our study is relevant. These authors demonstrated that there was no significant relationship between patient age and increased GSV diameter, particularly in patients classified as C0. Furthermore, they also concluded that increase in BMI is the most important factor in increased area, in agreement with the results found in the present study.

In our study, we assessed the relationship between GSV diameters and patients’ ages, since the prevalence of CVI tends to increase with age. Capitão et al.^10^ conducted an epidemiological study of CVI in Portugal and showed that class 3 CVI increases significantly after 50 years of age, irrespective of sex. Since it was necessary for our study to assess saphenous veins without reflux, patients free from advanced venous disease were recruited (clinical classes C0 to C2), and in this specific population we observed that normal GSVs do not change with age in people with mild to moderate CVI.

In conclusion, we identified diameters of GSVs without reflux of approximately 6.5 mm at the SFJ, 4.0 mm at the proximal thigh, 3.0 mm at the mid thigh, distal thigh, and knee, and 2.5 mm at the leg. Additionally, we concluded that these diameters are irrespective of CEAP clinical class 0/1 or 2, of age, and of height, but are related to patients’ BMI.
